# T-Cell Immunity to Infection with Dengue Virus in Humans

**DOI:** 10.3389/fimmu.2014.00093

**Published:** 2014-03-07

**Authors:** Daniela Weiskopf, Alessandro Sette

**Affiliations:** ^1^Division of Vaccine Discovery, La Jolla Institute for Allergy and Immunology, La Jolla, CA, USA

**Keywords:** DENV, T cells, protection, pathogenesis, HLA, vaccines

## Abstract

Dengue virus (DENV) is the etiologic agent of dengue fever, the most significant mosquito-borne viral disease in humans. Up to 400 million DENV infections occur every year, and severity can range from asymptomatic to an acute self-limiting febrile illness. In a small proportion of patients, the disease can exacerbate and progress to dengue hemorrhagic fever and/or dengue shock syndrome, characterized by severe vascular leakage, thrombocytopenia, and hemorrhagic manifestations. A unique challenge in vaccine development against DENV is the high degree of sequence variation, characteristically associated with RNA viruses. This is of particular relevance in the case of DENV since infection with one DENV serotype (primary infection) presumably affords life-long serotype-specific immunity but only partial and temporary immunity to other serotypes in secondary infection settings. The role of T cells in DENV infection and subsequent disease manifestations is not fully understood. According to the original antigenic sin theory, skewing of T-cell responses induced by primary infection with one serotype causes less effective response upon secondary infection with a different serotype, predisposing to severe disease. Our recent study has suggested an HLA-linked protective role for T cells. Herein, we will discuss the role of T cells in protection and pathogenesis from severe disease as well as the implications for vaccine design.

## World Wide Medical and Societal Significance of DENV and DENV Infection

Dengue virus (DENV) is the causative agent of dengue fever, the most prevalent mosquito-borne viral illness in humans and is primarily transmitted by the mosquitoes *Aedes aegypti* and *Aedes albopictus* (1). The world wide distribution of these two major vectors puts nearly a third of the global human population at risk of infection (2). It was recently reported that as many as 390 million dengue infections occur worldwide each year, thus making this infection potentially even more prevalent than malaria (3). Recent outbreaks in Europe (4) and the US (5, 6), led to the recognition of DENV as a Category A priority pathogen by NIAID and the classification of DENV as a domestic re-emerging disease threat by the CDC.

Disease can be induced by any of the four serotypes of DENV (DENV1–4). DENV-associated disease in most cases ranges from asymptomatic to an acute self-limiting febrile illness. However, in a small proportion of patients, the disease can exacerbate and progress to severe forms of dengue disease [dengue hemorrhagic fever (DHF) and dengue shock syndrome (DSS)], characterized by severe vascular leakage, thrombocytopenia, and hemorrhagic manifestations (7). Infection with one DENV serotype presumably results into life-long immunity to the infecting serotype but does only confer short-term protection against the other serotypes (8). In fact, the severe forms of DENV disease are more often observed in individuals experiencing a secondary infection with a different serotype (9, 10). Besides prior infection with one serotype, other factors influencing the disease outcome are the strain of infecting virus, age and gender, nutritional status, and the genetic background of the patient (11–16). No licensed vaccine or effective anti-viral therapy is currently available and treatment is largely supportive in nature, thus increasing the economic and disease burden on public health systems in tropical and subtropical countries around the globe (17–19).

## DENV-Specific T-Cell Responses

### T-cell epitope identification

A previous analysis (20) documented the substantial knowledge gaps existing at the level of defined T- and B-cell immune responses. Over the last years, the situation has improved considerably. As of June 2013, the immune epitope database (IEDB)^1^ lists 369 antigenic regions identified in humans and 71 identified in HLA transgenic mice. It has been shown that CD8^+^ T cells most vigorously and frequently recognized the NS3, NS4B, and NS5 proteins, whereas the capsid, envelope, and NS3 proteins are the dominant targets for CD4^+^ T cells (21–25). In parallel, but beyond the scope of this review, significant strides have been made in the definition of DENV-related B cell epitopes.

Despite these efforts, significant gaps yet remain. First, the vast majority of DENV epitopes described in the literature and reported in the IEDB are restricted by HLA MHC class I alleles, and only 13% of the epitopes are restricted by HLA class II and recognized by CD4^+^ T cells. Of those epitopes, only a few have defined allele and loci restrictions and characterized functional and phenotypic features of the associated T cells. Thus, a comprehensive analysis of MHC class II restricted responses across all loci is needed in the general population from endemic areas and in patient populations associated with different severity of disease (acute DF versus DHF/DSS). Furthermore, the epitopes recognized after vaccination with experimental vaccines have not been systematically identified or validated. This hampers progress in the field, as the role of MHC class I and class II restricted responses in disease protection and immunopathology cannot be broadly evaluated, and the performance of different vaccines in terms of induction of immune responses in human vaccines remains undetermined.

### Implications for HLA polymorphism

T cells recognize a complex of a particular pathogen-derived epitope presented by a specific MHC molecule. Thus, a given epitope will elicit a response in individuals that express MHC molecules capable of binding that particular epitope. MHC molecules are extremely polymorphic, with several thousand variants known in humans (26, 27). Each variant is present with variable frequency, depending on ethnic lineage and geographic locality. As a result, for basic investigations, diagnostic or vaccine applications and to ensure high and non-ethnically biased coverage of different patient populations, it is necessary that the alleles investigated are carefully selected. This is accomplished by selecting those alleles that are most frequent in the various population groups worldwide.

To address this challenge in the context of HLA class I, we have applied a selection process focused on the 27 most common HLA A and B alleles in the general population (25). As previously described, these 27 alleles allowed us to cover at least three out of four possible HLA A and B alleles expressed per donor in 90% of a cohort from the general population of Colombo, Sri Lanka. In the case of HLA class II, we have recently reported the selection of a panel of 27 different allelic variants that affords high coverage of all four HLA class II loci (DRB1, DRB3/4/5, DQ, and DP) (27). Based on publically available population frequency data (28), this panel of HLA DR, DQ, and DP specificities should allow to cover over 98% of individuals in the general population. Notably, the actual coverage achieved by this panel was similarly high in cohorts of distinctly different ethnic composition that we have previously utilized for our studies (29, 30). For each of these class II molecules, we have established quantitative binding assays (27) and generated a sufficiently large number of measurements to enable derivation of quantitative algorithms for predicting binding capacity (27, 31). Predictive algorithms for the most common HLA class I and class II alleles are now publicly available at several web sites, including the IEDB^2^. Additionally, for each molecule we have produced cell lines transfected with a single HLA class II allele that will be useful for fine mapping of HLA restrictions (30). These approaches now represent efficient and valuable tools for epitope identification, especially in the context of large and complex targets.

### The hypothesis of original antigenic sin as it is related to a potential role of T cells in DENV pathogenesis

It has been proposed that cross-reactive T cells raised against the original infecting serotype dominate during a secondary heterologous infection, a phenomenon that has been termed “original antigenic sin” (32, 33). This term was first applied to the humoral response to influenza epidemics (34), but has also been observed in CD8^+^ T-cell responses against lymphocytic choriomeningitis virus (LCMV) (35). This hypothesis postulates that during secondary infection, expansion of pre-existing lower avidity cross-reactive memory T cells dominate the responses over that of naïve T cells that are of higher avidity for the new DENV serotype. It is further hypothesized that peptide variants derived from the secondary infection serotype can induce a response that is qualitatively different from the response induced by the original antigen, such as inducing a different pattern of cytokine production. Variants associated with this phenotype are often collectively referred to as altered peptide ligands (APLs) (36). It is hypothesized that these altered T-cell responses serotype may contribute to a “cytokine storm” during heterologous secondary infection and thus contribute to immunopathogenesis of severe dengue disease (33). However, this hypothesis is in conflict with the observation that heterologous T-cell responses are not always needed to produce severe disease in infants. DHF or DSS in infants generally occurs between the ages of 6 and 12 months in endemic areas (37). When the maternal antibody titer to DENV decreases below a protective level, infants are actually at an increased risk for the development of severe disease despite the fact that they have never been infected with DENV and lack DENV-specific T-cell memory (38). Furthermore, a recent study has shown a temporal mismatch between the CD8^+^ T-cell response and commencement of capillary leakage, suggesting that CD8^+^ T cells are not responsible for early triggering of capillary leakage in children with DHF (39).

We have previously reported that “original antigenic sin” is indeed detectable at the level of CD8^+^ T-cell responses in the general population (25). However, a potential limitation of those studies was that they were conducted at the level of the general population from an endemic area (i.e., Sri Lanka), and did not measure HLA class II restricted epitopes. Furthermore, it is not known whether the studies could capture *in vivo* impaired or altered T-cell responses during acute infection.

### Low magnitude T-cell responses are HLA-linked and associated with disease susceptibility

The results presented above suggest that antigenic sin does not significantly impair the quality of T-cell responses in the general population. However, lower quality responses might be associated with the relatively few individuals experiencing more severe clinical outcomes. Previous studies highlight that certain HLA alleles are associated with either increased or decreased risk of clinical manifestations (14, 40–45). However, these studies did not determine whether increased risk might be associated with a hyperactive T-cell response, or conversely whether a higher T-cell response might be associated with a decreased risk. Correlations of HLA-associated disease susceptibility with T-cell responses found that weak T-cell responses correlated with disease susceptibility (25). A possible explanation for these observations would be that certain alleles and epitopes are associated with higher magnitude responses, which are in turn associated with higher degrees of multi-functionality, and thus might be most beneficial in protecting from disease. A detailed analysis of cytokines produced by DENV-specific T cells revealed that stronger responses are indeed associated with multifunctional T-cell responses. Thus, it might be possible that while T cells have a protective role in general in the HLA-linked, lack of a multifunctional T-cell response might contribute to pathogenesis in certain individuals.

### Role of T cells in protection against DENV infection

The protective role of T cells during viral infection is well established (46). Generally, CD8^+^ T cells can control viral infection through several mechanisms, including direct cytotoxicity and production of pro-inflammatory cytokines such as IFN-γ and TNFα. Similarly, CD4^+^ T cells are thought to control viral infection through multiple mechanisms, including enhancement of B and CD8^+^ T-cell responses, production of inflammatory and anti-viral cytokines, cytotoxicity of viral infected cells, and promotion of memory responses (47).

Several lines of evidence suggest that both CD4^+^ and CD8^+^ T cells may contribute to protection against homologous re-infection or heterologous dengue infection. It has been shown that DENV-specific human CD4^+^ T and CD8^+^ T cells proliferate, produce IFN-γ, and lyse infected target cells (48–50), suggesting that serotype-specific T cells are activated and functional in humans with primary DENV infection (48, 51). Furthermore, higher frequencies of DENV-specific IFNγ-producing T cells are present in children who subsequently develop subclinical infection, compared with those who develop symptomatic secondary DENV infection (52).

Finally, studies in a murine model of DENV infection demonstrated that both CD4^+^ and CD8^+^ T cells contribute to protection against DENV challenge (53–56). In parallel to the evidence in the murine model, studies performed previously demonstrated that HLA alleles associated with increased risk of severe disease are also associated with weak CD8^+^ T-cell responses, and conversely that strong, multifunctional, T-cell responses correlate with alleles associated with protection from severe disease. These data strongly imply a protective role for CD8^+^ T cells against severe DENV disease in humans (25).

## DENV Serotypes and Vaccine Development

The dengue serocomplex consists of four serotypes, each of which is made up of several genotypes (57). The four serotypes share 65–75% genetic homology with each other but are antigenically distinct (58). This high degree of sequence variation in a pathogen, characteristically associated with RNA viruses, poses unique challenges to vaccine development. This is of particular relevance in the case of DENV infections because of the more severe disease and immunopathology associated with prior exposure to a different serotype (9). Consequently, the development of DENV vaccines has been hampered by the potential risk of vaccine-related adverse events and the requirement to induce long-lasting protective immune responses against all four DENV serotypes simultaneously. A recent phase 2b proof-of-concept efficacy vaccine trial (59) of a live-attenuated tetravalent chimeric yellow fever-dengue vaccine (CYD23) showed only 30% overall efficacy, demonstrating partial (60–80%) protection toward three of four DENV serotypes. No protection against DENV2 infection was observed, despite three subsequent immunizations and high neutralization titers against all four serotypes.

As reviewed above, T-cell responses have been implicated to have a protective role in DENV infection. Previous data from our lab and others clearly demonstrate that CD8^+^ T-cell responses dominantly target the non-structural proteins NS3, NS4B, and NS5 (21–25). Since these DENV proteins are absent in the recombinant live-attenuated tetravalent dengue-yellow fever chimeric virus vaccine (60), our results perhaps provide an explanation for the low level of vaccine efficacy observed. Further, our data demonstrate the need to accurately assess T-cell responses (and not only antibody responses) in the context of DENV vaccine development.

Five additional and promising vaccine candidates are being tested in human clinical trials. These vaccines rely on technologies spanning from live-attenuated viruses, vectored vaccines expressing certain dengue proteins, replication-defective vaccines to nucleic acid-based vaccines [reviewed in Ref. (61)]. Since our data raise the possibility that T-cell responses against all DENV proteins might be beneficial or even required for vaccine efficacy, it will be of particular interest to study T-cell epitopes induced by multivalent live-attenuated vaccines and compare them to T-cell responses observed in natural infection.

### Methods to characterize T-cell responses after vaccination

Characterization of T-cell epitopes can be performed by a variety of techniques, each associated with distinct advantages and disadvantages. These techniques include: ELISPOT, FACS and ICS assays, cell sorting, and tetramer staining. Though ELISPOT is the most sensitive at detecting low-levels of specific cytokine production, ICS assays are better suited to characterize phenotypes and T cells that are simultaneously producing multiple cytokines. Secretion of particular cytokines such as IFN-γ and TNF-α has been implicated in the induction of DENV-associated immunopathology. IFN-γ has been implicated as having a protective role during DENV infection whereas TNF-α has been implicated as a key mediator of immunopathology (62, 63). Characterization of a broad array of cytokines affords determination of the degree to which the cells responding to a given epitope are polyfunctional effectors. As illustrated in several different systems, T cells with a polyfunctional phenotype capable of secreting multiple cytokines provide the most effective control of viral infection (64–66). Importantly, both ELISPOT and ICS assays can be used to characterize pools of epitopes in conditions where only small amounts of PBMC are available.

An alternative and complementary approach to ELISPOT and ICS involves the use of tetramer staining reagents (67, 68). This approach requires not only the production of specific reagents for each HLA:epitope combination, but also that T cells specific for each combination are present in sufficient frequency in peripheral blood to allow their detection and characterization. In cases where T-cell frequency is low, this limitation can be overcome by the tetramer enrichment technique (69). Because tetramer characterization is in general more technically demanding, tetramer assays are ideally suited for in-depth characterization of a small but representative set of epitope specificities.

Markers associated with memory or activation/exhaustion states are also of interest. For vaccines to be effective, they must promote development of an effective T-cell memory response, in terms of recall of both effector T-cell responses and anamnestic antibody responses (70). T cell can be classified into T_N_ (naïve T cells), T_CM_ (central memory T cells), T_EM_ (effector memory T cells), and T_EMRA_ (effector memory T cells re-expressing CD45RA) subsets (71). Activation and conversely exhaustion of T cells are implicated in the regulation of protective immunity and immunopathology (72). Markers such as CD57 (73, 74), CD40L (75), and PD-1 (programed death-1) (76) allow determination of activation/exhaustion states of memory T-cell subsets. All of these techniques are currently available and a detailed analysis of the responses induced by natural or experimental infection with DENV will greatly contribute to the understanding of T-cell immunity in humans and may further contribute to identify robust correlates of protection in natural immunity and vaccination against DENV.

## Conflict of Interest Statement

The authors declare that the research was conducted in the absence of any commercial or financial relationships that could be construed as a potential conflict of interest.

## Footnotes

^1^www.iedb.org

^2^www.immuneepitope.org
